# *TUFT1*, a novel candidate gene for metatarsophalangeal osteoarthritis, plays a role in chondrogenesis on a calcium-related pathway

**DOI:** 10.1371/journal.pone.0175474

**Published:** 2017-04-14

**Authors:** Eeva Sliz, Mari Taipale, Maiju Welling, Sini Skarp, Viivi Alaraudanjoki, Jaakko Ignatius, Lloyd Ruddock, Ritva Nissi, Minna Männikkö

**Affiliations:** 1 Center for Life Course Health Research, Faculty of Medicine, University of Oulu, Oulu, Finland; 2 Faculty of Biochemistry and Molecular Medicine, University of Oulu, Oulu, Finland; 3 Biocenter Oulu, University of Oulu, Oulu, Finland; 4 Medical Research Center Oulu, Oulu University Hospital and University of Oulu, Oulu, Finland; 5 Research Unit of Oral Health Sciences, University of Oulu, University of Oulu, Oulu, Finland; 6 Department of Clinical Genetics, Turku University Hospital, Turku, Finland; 7 Department of Obstetrics and Gynecology, Oulu University Hospital, Oulu, Finland; Instituto Butantan, BRAZIL

## Abstract

Osteoarthritis (OA) is the most common degenerative joint disorder and genetic factors have been shown to have a significant role in its etiology. The first metatarsophalangeal joint (MTP I) is highly susceptible to development of OA due to repetitive mechanical stress during walking. We used whole exome sequencing to study genetic defect(s) predisposing to familial early-onset bilateral MTP I OA inherited in an autosomal dominant manner. A nonsynonymous single nucleotide variant rs41310883 (c.524C>T, p.Thr175Met) in *TUFT1* gene was found to co-segregate perfectly with MTP I OA. The role of *TUFT1* and the relevance of the identified variant in pathogenesis of MTP I OA were further assessed using functional *in vitro* analyses. The variant reduced *TUFT1* mRNA and tuftelin protein expression in HEK293 cells. ATDC5 cells overexpressing wild type (wt) or mutant *TUFT1* were cultured in calcifying conditions and chondrogenic differentiation was found to be inhibited in both cell populations, as indicated by decreased marker gene expression when compared with the empty vector control cells. Also, the formation of cartilage nodules was diminished in both *TUFT1* overexpressing ATDC5 cell populations. At the end of the culturing period the calcium content of the extracellular matrix was significantly increased in cells overexpressing mutant *TUFT1* compared to cells overexpressing wt *TUFT1* and control cells, while the proteoglycan content was reduced. These data imply that overexpression of *TUFT1* in ATDC5 inhibits chondrogenic differentiation, and the identified variant may contribute to the pathogenesis of OA by increasing calcification and reducing amount of proteoglycans in the articular cartilage extracellular matrix thus making cartilage susceptible for degeneration and osteophyte formation.

## Introduction

Osteoarthritis (OA) is one of the most common musculoskeletal disorders worldwide and its prevalence is predicted to increase in the future [1]. OA is a disease of the whole joint [2] and the main pathologic changes are progressive loss of articular cartilage, joint space narrowing, osteophyte formation, subchondral bone sclerosis, and cyst formation [3]. These lead to pain and loss of joint function in OA patients [4]. During the development of OA chondrocytes start to proliferate and express matrix-degrading enzymes leading to matrix remodeling involving hypertrophic maturation of chondrocytes and calcification of cartilage [5]. In general, OA is considered as a complex trait caused by interplay between genetic and environmental factors [6]. In twin studies the influence of genetic components has been predicted to be as high as 39–65% depending on joint site [7]. Although OA generally lacks a clear Mendelian pattern of inheritance, rare familial early-onset forms with autosomal dominant inheritance have been described [8–11].

Foot OA is poorly studied in comparison to hip or knee OA, although the first metatarsophalangeal (MTP I) joint is often affected by OA [12]. The prevalence of radiographic MTP I OA has been estimated to be from 6.3% to 39% in middle-aged and older adults [13]. Foot OA shares many risk factors with other forms of OA, like age and obesity. Also, mechanical stress, trauma and inflammation are often associated with foot OA [12]. Individuals with symptomatic MTP I OA experience localized pain and stiffness during movement and therefore have difficulties in various physical tasks such as walking [14].

Nissi *et al*. (2011) reported a family with early-onset bilateral foot arthritis limited to the MTP I joint [15]. The family history strongly suggests an autosomal dominant inheritance. In order to identify the genetic defect(s) predisposing to this disorder, we performed whole exome sequencing on the aforementioned family and identified a variant in *TUFT1* co-segregating with the disease. *TUFT1*, encoding tuftelin protein, is previously known to be involved in enamel mineralization [16] and is recognized as a candidate gene for dental caries [17], but is suggested to have a universal or multifunctional role due to its expression in multiple cell and tissue types [18–20]. In cartilage, *TUFT1* expression is strongest in the deeper, mineralizing zones [21]. Interestingly, *TUFT1* expression has been shown to be regulated *in vitro* by hypoxia-inducible factor 1-alpha and hedgehog pathways, both essential for cartilage and bone formation [22,23]. However, the precise function of tuftelin is not fully known. We show that *TUFT1* participates in regulation of chondrocyte differentiation and that the identified variant gives rise to extracellular matrix (ECM) modifications observed in association with OA. We propose that *TUFT1* is a novel candidate gene for foot OA and that the identified variant is likely to be involved in the pathogenesis of foot OA in the studied family.

## Results

### Whole exome sequencing

**Fig 1:**
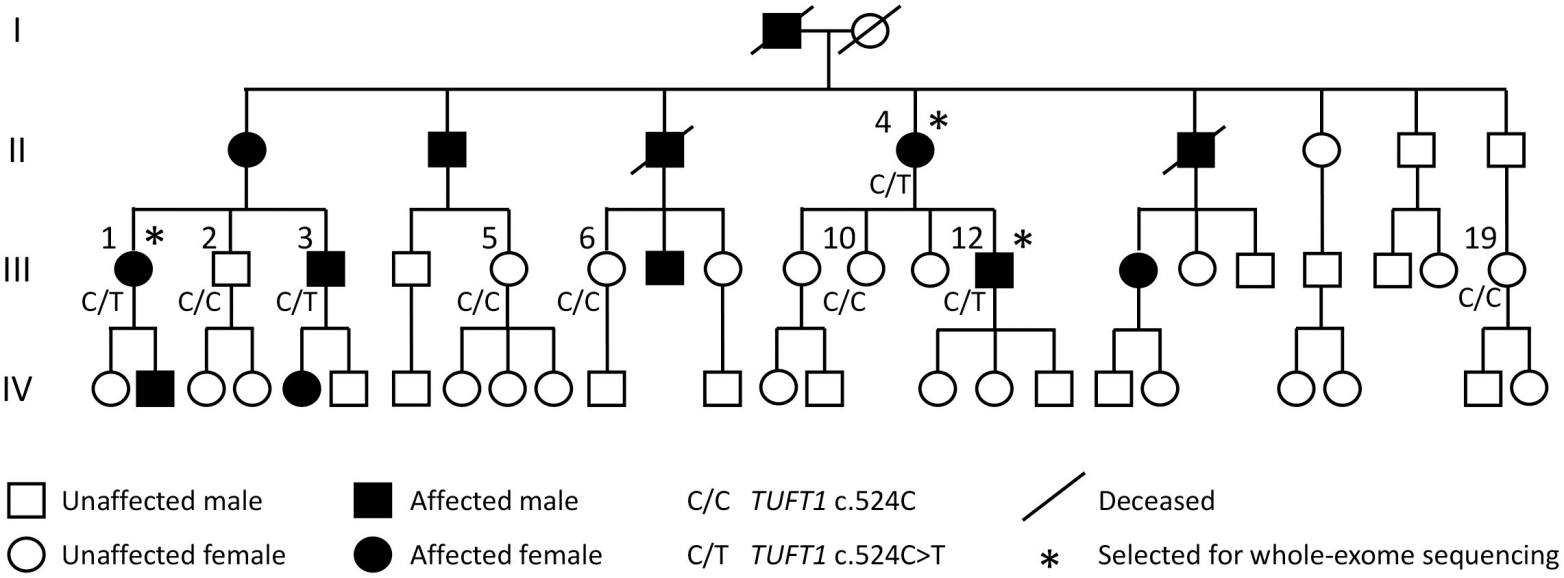
The pedigree of the Finnish MTP I OA family and the *TUFT1* c.524C>T segregation. The numbering indicates individuals whose DNA was available for the study. The *TUFT1* genotype is indicated below each individual. The pedigree is modified from Nissi *et al*. 2011 [15].

Three affected individuals from a Finnish family with MTP I OA (Fig 1) and two unrelated individuals (controls) were analyzed using whole exome sequencing. Summary of the exome sequencing and variant annotation is shown in Table 1. The three patients shared in total 48 860 single nucleotide variants (SNVs) and 4 670 insertions and deletions (indels). Following the variant filtering steps, altogether 33 SNVs and five indels (S1 Table) were selected for validation by Sanger sequencing in all nine family members whose DNA was available for the study (four affected, five unaffected, Fig 1). The variant rs41310883 (c.524C>T) in *TUFT1* was found heterozygous in all four patients, but was not detected in healthy family members, being the only variant perfectly co-segregating with the MTP I OA in the family. The variant introduces a threonine to methionine substitution at position 175 (p.Thr175Met, NP_064512).

**Table 1:** Summary statistics for exome sequence data analysis steps.

	SNV count	Indel count
Autosomal variants shared by the three affected family members	48 860	4 670
Shared by the affected family members, not found in the controls	5 794	525
Novel and rare variants (MAF≤0.01 or unknown)	739	366
Exonic or splicing	143	12
Harmful *in silico* prediction	33	5
Co-segregates with MTP I OA*	1	0

SNV, single nucleotide variant; indel, insertions and deletions,

*validation by Sanger sequencing in nine family members.

### Detection of Copy Number Variations (CNVs)

CNVs calls were generated from exome data to screen possible structural genome variations such as deletions and duplications. Altogether 160 CNVs were called in chromosomes 1–22. Of these, 142 were deletions and 18 were amplifications. The three affected family members had a mutual CNV region on chromosome 17 where two patients (II-4 and III-1) showed a heterozygous deletion and one patient (III-2) had an amplification (data not shown). Annotation to the Database of Genomic Variants showed that this region has been previously reported to be a common CNV region [24].

### The effect of c.524C>T on mRNA and protein expression

**Fig 2:**
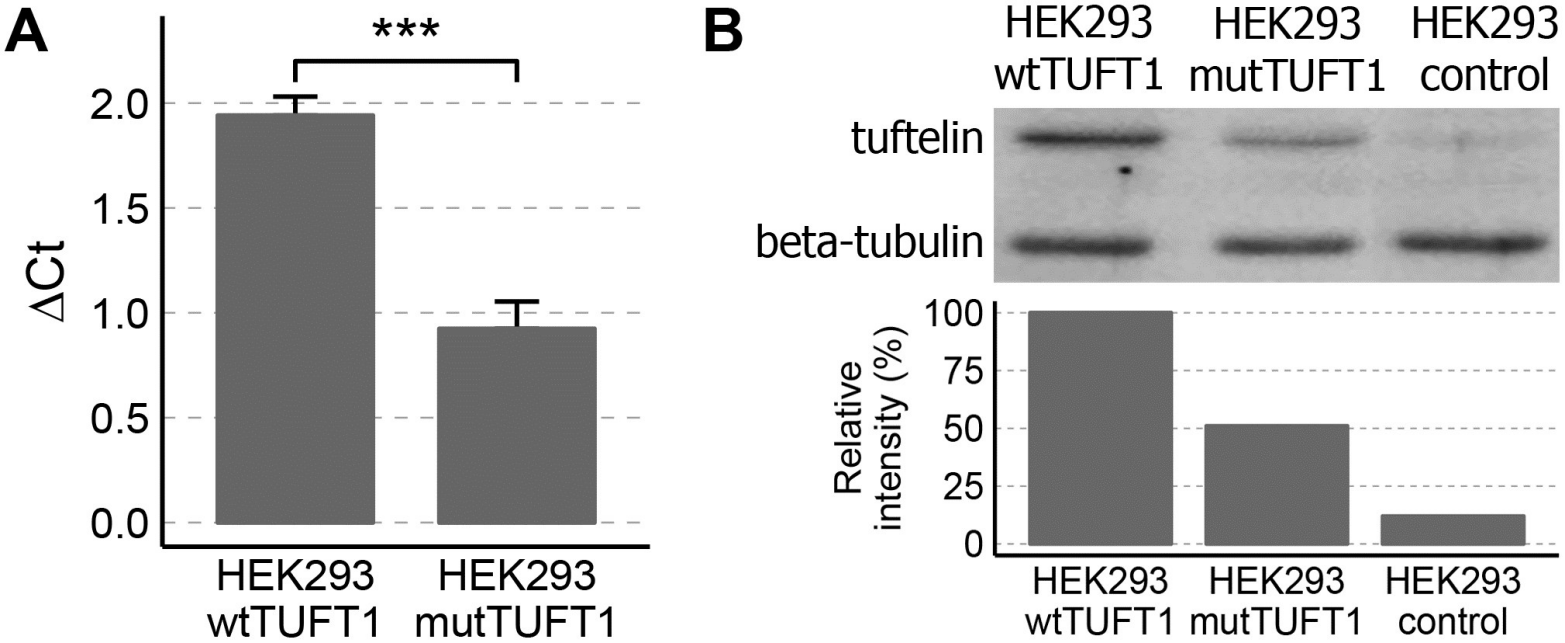
The effect of the c.524C>T variant on *TUFT1* expression in *TUFT1* overexpressing HEK293 cells. (A) *TUFT1* mRNA expression in HEK293-mutTUFT1 cells was approximately 47% of the expression observed in HEK293-wtTUFT1 cells (*** P ≤ 0.001). *ACTB* and *B2M* were used as reference genes. (B) The variant suppressed also tuftelin protein expression, as the relative intensity of tuftelin in HEK293-mutTUFT1 cells was 51% of the relative intensity of tuftelin observed in HEK293-wtTUFT1 cells. The relative intensities were calculated from the ratio of tuftelin to beta-tubulin absolute intensities. The faint band in the control cells corresponds to the endogenous tuftelin recognized by the antibody.

The function of the identified variant was studied *in vitro* in human embryonic kidney (HEK293) cells. Cells transfected with the mutant *TUFT1* construct (HEK293-mutTUFT1) generated less (P ≤ 0.001) *TUFT1* mRNA than cells transfected with the wt *TUFT1* construct (HEK293-wtTUFT1, Fig 2A). Consistent with this, the variant attenuated tuftelin protein expression (Fig 2B and S1 Fig).

### The role of *TUFT1* overexpression in chondrocyte differentiation

To study the functional role of tuftelin in chondrogenesis and mineralization, ATDC5 cells stably overexpressing wt or mutant tuftelin (ATDC5-wtTUFT1 or ATDC5-mutTUFT1, respectively) and empty vector controls (ATDC5-ctrl) were grown in calcifying conditions in the presence of insulin, ascorbic acid and beta-glycerophosphate (βGP) for 15 days. Expression of three marker genes for chondrogenic differentiation, sex determining region Y-box 9 (*Sox9*), type II collagen (*Col2a1*), aggrecan (*Agc1*), and four marker genes for chondrocyte hypertrophy, runt-related transcription factor 2 (*Runx2)*, type X collagen (*Col10a1*), matrix metallopeptidase *13 (Mmp13*), and alkaline phosphatase (*Alpl*) were studied at three time points using real-time quantitative polymerase chain reaction (qPCR).

**Fig 3:**
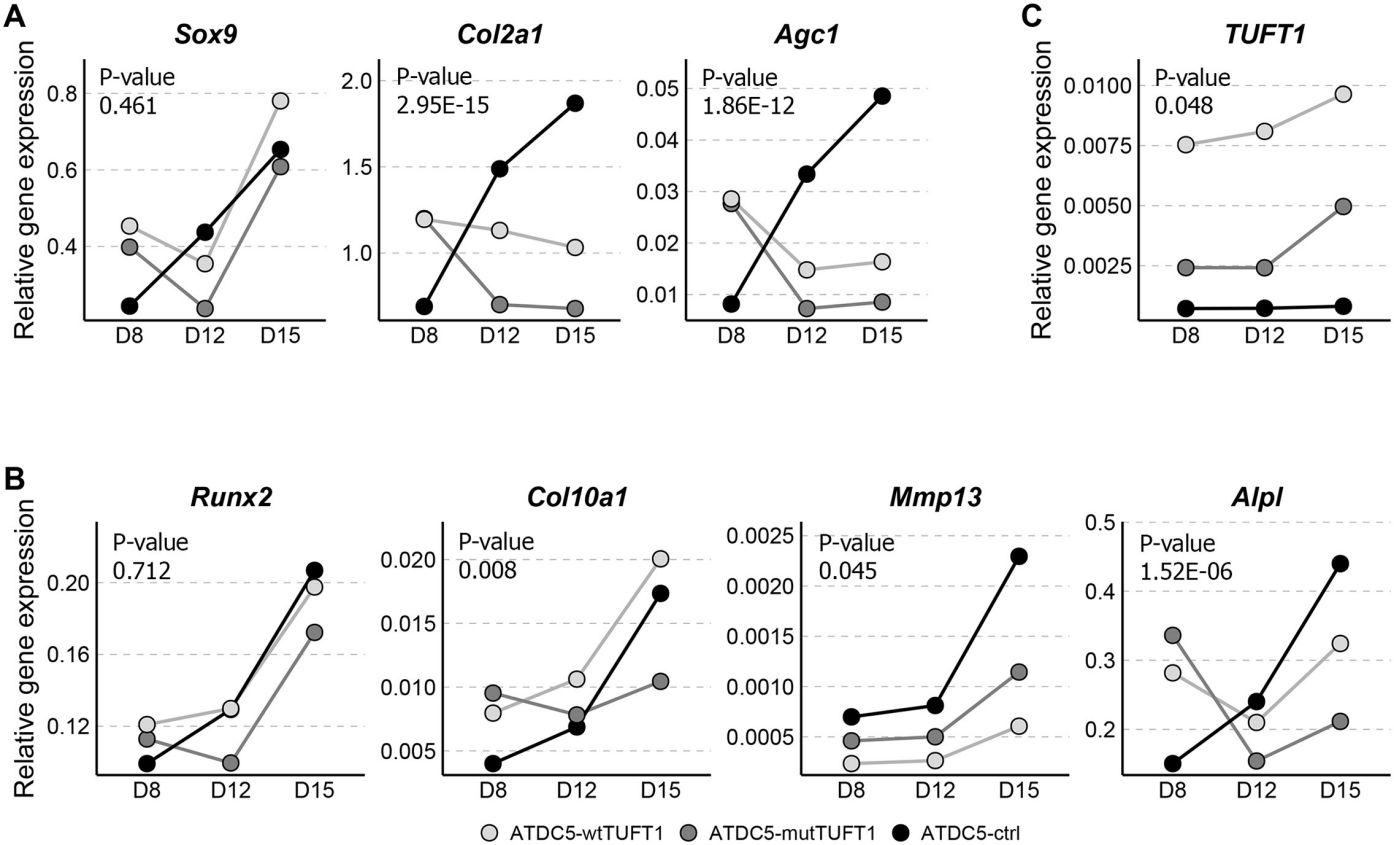
Expression of chondrocyte differentiation and hypertrophy marker genes, and *TUFT1* in ATDC5 cells. (A) *Sox9*, *Col2a1*, and *Agc1* are markers for chondrocyte differentiation and (B) *Runx2*, *Col10a1*, *Mmp13*, and *Alpl* markers for chondrocyte hypertrophy. In addition, expression of *TUFT1* was determined (C). Expressions were studied by real-time qPCR in mixed population clones overexpressing wt or mutant *TUFT1* (ATDC5-wtTUFT1, ATDC5-mutTUFT1) and in empty vector controls (ATDC5-ctrl) at three time points during 15 days of differentiation. *Hprt* and *Ppia* were used as reference genes. Results are represented as means of three groups of quadruplet samples and relative gene expression denotes log(x+1) transformed fold changes. P-values relate to the confidence of whether the change in the relative gene expression over time is different between the ATDC5-wtTUFT1, ATDC5-mutTUFT1 and ATDC5-ctrl cell populations (S2 Table).

Overexpression of both wt and mutant *TUFT1* significantly influenced the expression of *Col2a1* and *Agc1* (P = 2.95x10^-15^ and P = 1.86x10^-12^, respectively, Fig 3A, S2 Table), but did not affect the expression of *Sox9* (P = 0.461, Fig 3A, S2 Table): in ATDC5-mutTUFT1 cells *Col2a1* expression differed from control cells on all the measurement days whereas in ATDC5-wtTUFT1 cells the difference reached statistical significance on days 8 and 15 (S3 Table). Additionally, *Col2a1* expression was significantly lower in ATDC5-mutTUFT1 cells in comparison to ATDC5-wtTUFT1 cells on days 12 and 15 (P = 0.008 and P = 0.045, respectively, Fig 3A, S3 Table). *Agc1* expression in both ATDC5-mutTUFT1 and ATDC5-wtTUFT1 cells deviated from the expression seen in control cells, while there was no statistically significant difference between the mutant and wt cells (S3 Table).

No statistically significant differences were observed in the *Runx2* expression on any of the days (Fig 3B, S3 Table). *Col10a1* expression showed a similar trend in ATDC5-ctrl and ATDC5-wtTUFT1 cells. On day 15 expression of *Col10a1* in the ATDC5-mutTUFT1 cells was significantly lower when compared to the ATDC5-wtTUFT1 cells (P = 0.007, Fig 3B, S3 Table). The expression of *Mmp13* was significantly lower in both ATDC5-wtTUFT1 and ATDC5-mutTUFT1 cells in comparison to the control cells on day 15 (P = 2.09x10^-5^ and P = 0.011, Fig 3B, S3 Table). The expression of *Alpl* in ATDC5-mutTUFT1 cells was significantly higher in control cells already on day eight (P = 0.005, Fig 3B, S3 Table), whereas on day 15 the *Alpl* expression in ATDC5-mutTUFT1 cells was significantly lower than in control cells (P = 2.12x10^-4^, Fig 3B, S3 Table).

Expression of *TUFT1* was assessed at three time points (Fig 3C). Comparable with the study done in HEK293 cells, *TUFT1* expression was lower in the ATDC5-mutTUFT1 cells than in the ATDC5-wtTUFT1 cells at all time points. All three cell populations showed an increasing trend of *TUFT1* expression during the 15-day period. Results of gene expression studies in ATDC5 cells are presented in Fig 3, and the test statistics are summarized in S2 and S3 Tables.

### Contribution of *TUFT1* overexpression to extracellular matrix mineralization and nodule formation

**Fig 4:**
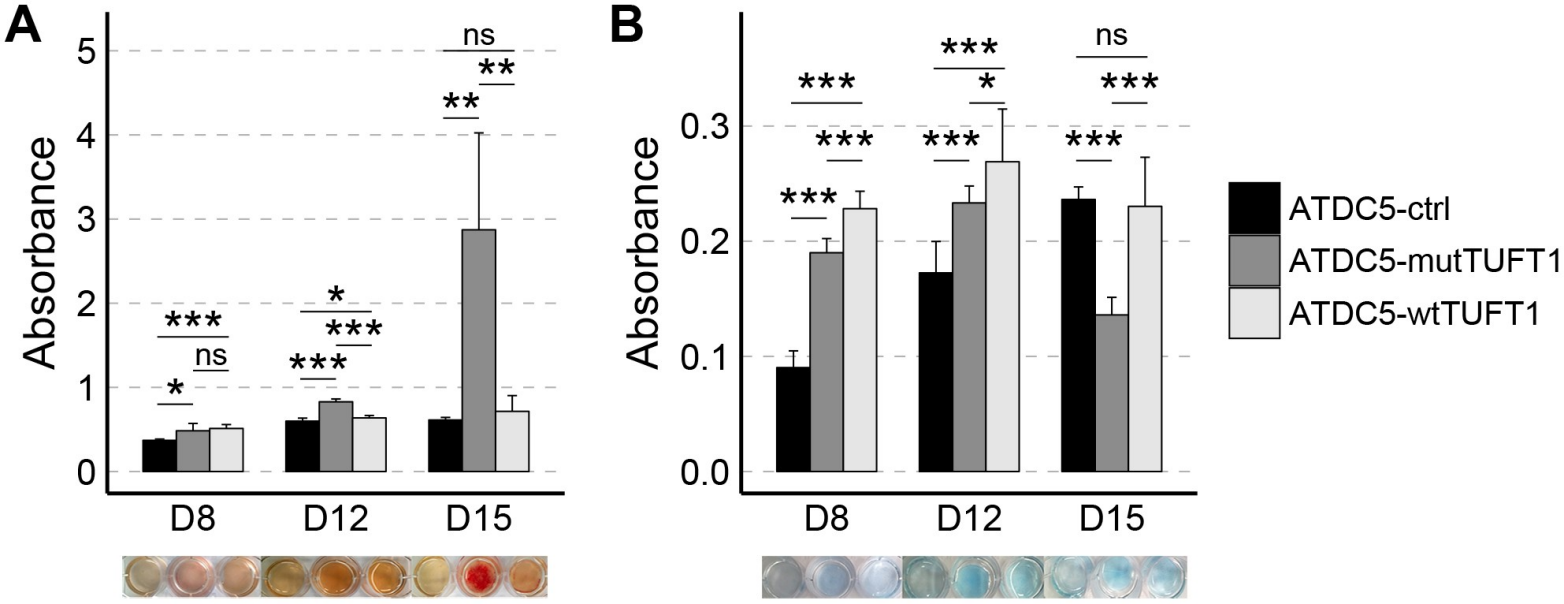
ECM mineralization in ATDC5-ctrl, ATDC5-mutTUFT1 and ATDC5-wtTUFT1 cells during differentiation. (A) Calcium content was visualized by Alizarin red staining and (B) proteoglycan content by Alcian blue staining. The staining was quantified by spectrophotometer at 570 nm and 630 nm, respectively. Measurements were performed on the days 8, 12 and 15 and results are represented as mean ± standard deviation of four wells analyzed. Abbreviations: ns, not significant; * P ≤ 0.05; ** P ≤ 0.01; *** P ≤ 0.001.

Calcium and proteoglycan content in the extracellular matrix (ECM) of the ATDC5 cells were studied at three time points along the 15-day culture period using Alizarin red and Alcian blue stainings, respectively. In general, the contents of both calcium (Fig 4A) and proteoglycans (Fig 4B) in the ECM of both ATDC5-mutTUFT1 and ATDC5-wtTUFT1 cells deviated from what was seen in control cells at the three time points. The most evident deviation was the significant increase in the calcium content in the ECM of ATDC5-mutTUFT1 cells on day 15 (Fig 4A). On the contrary on day 15 the ATDC5-mutTUFT1 cells showed reduced amount of proteoglycans in the ECM when compared with the control and ATDC5-wtTUFT1 cells (Fig 4B).

**Fig 5:**
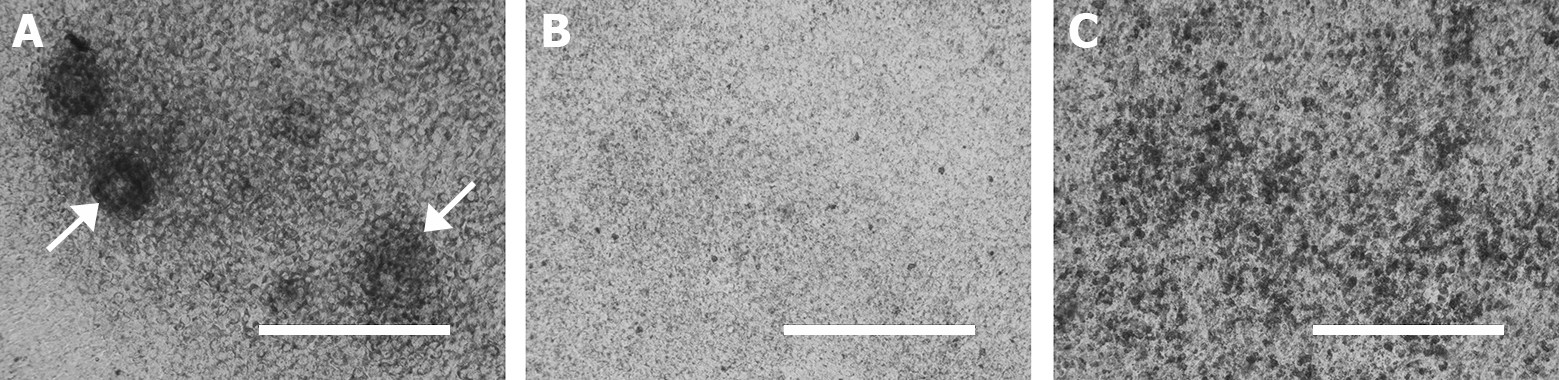
Formation of mineralizing cartilage nodules in ATDC5 cells overexpressing *TUFT1 in vitro*. (A) Control cells formed mineralizing cartilage nodules (arrow), seen as centred opaque deposits in the microscopic images taken on day 15 of differentiation. (B) Nodule and mineralization center formation was diminished in ATDC5-wtTUFT1 cells. (C) Nodule formation was impaired also in ATDC5-mutTUFT1 cells and mineralization occurred in a disordered manner throughout the culture. The scale bar represents 400 μm.

During the differentiation experiment, control cells begin to form cartilaginous nodules and ECM mineralization centres, seen as concentrated black deposits in Fig 5A. Nodule formation in ATDC5-wtTUFT1 cells was impaired when compared with control cells, and no mineralization centres were observed (Fig 5B). Nodule formation was also impaired in the ATDC5-mutTUFT1 cells and mineralization occurred disorderly throughout the cell culture (Fig 5C) compatible with the observed increase in the ECM calcium (Fig 4A).

## Discussion

In the present study we identified a rare nonsynonymous variant (rs41310883, c.524C>T, p.Thr175Met) in *TUFT1* that co-segregates with MTP I OA in a Finnish family. *TUFT1* encodes tuftelin protein which plays a role in enamel mineralization [16,17], but is also suggested to have a more universal function due to its wide expression in multiple cell and tissue types [18–20]. Our *in vitro* experiments showed that the c.524C>T variant decreased both *TUFT1* mRNA and tuftelin protein expression in *TUFT1* overexpressing HEK293 cells, and altered the calcium and proteoglycan content in the ECM of ATDC5 cells. Interestingly, overexpression of both wt and mutant *TUFT1* in ATDC5 cells altered chondrogenic differentiation, as indicated by atypical expression of differentiation marker genes and diminished formation of cartilage nodules. Our findings suggest that *TUFT1* plays a role in chondrocyte differentiation and cartilage mineralization apparently on a calcium-related pathway, and the identified variant likely contributes to the disease phenotype in the studied family by giving rise to cartilage ECM modifications often seen in association with OA.

The identified c.524C>T variant was found to alter the amount of calcium and proteoglycans in the ECM of ATDC5 cells over a 15-day culture period suggesting that the mutation alters cell functions involved in the assembly of ECM. Particularly the highly increased extracellular calcium on day 15 was the distinguishable feature that seemed to be due to the c.524C>T variant rather than due to *TUFT1* overexpression. The variant introduces an amino acid residue that is larger in size and more hydrophobic than the wt residue likely disrupting proper folding of the protein [25]. The mutation also resides in one of the two coiled-coil domains in tuftelin which may also contribute to altered function of tuftelin, as coiled-coil domains are important for proper protein folding and known to be crucial in multiple biological functions [26]. Furthermore, the mutation locates on one of tuftelin’s ten evolutionarily conserved phosphorylation sites that are predicted to function in chelation of calcium [27]. The observed effect of the mutation could be due to tuftelin’s altered affinity to calcium or due to improper folding disturbing protein-protein interactions or due to both of these. Increased calcification and degraded proteoglycans are distinctive features of osteoarthritic cartilage [5,28], and therefore it seems likely that the identified mutation is involved in the pathogenesis of the MTP I OA in the studied family.

Seemingly *TUFT1* overexpression alone is sufficient to interfere with chondrogenesis, as we discovered that in addition to ATDC5-mutTUFT1 cells chondrogenic differentiation was diminished also in ATDC5-wtTUFT1 cells. Both of the cell populations showed a decreasing trend of chondrogenic differentiation marker genes *Col2a1* and *Agc1* during the 15-day culture period while in the control cells the expression strengthened over time, as expected. In addition, in the beginning of chondrogenesis mesenchymal precursor cells should start to condensate to form nodules [29], whereas we observed that the nodule formation was diminished in both of the *TUFT1* overexpressing ATDC5 cell populations. This advocates that proper timing and/or quantity of *TUFT1* expression is crucial for the differentiation to proceed appropriately. Similar findings were obtained in a study done on mice, which indicated that tuftelin overexpression disturbed the growth of carbonated calcium hydroxyapatite crystallites in developing enamel and the phenotype was most evident in mice with the greatest tuftelin expression [30].

Tuftelin appears to function at the very beginning of the differentiation process: we observed that the differentiation marker gene expression as well as the ECM mineralization in both ATDC5-mutTUFT1 and ATDC5-wtTUFT1 cells deviates from what is seen in the control cells already on day eight. A highly similar phenotype with impaired marker gene expression and diminished nodule formation during differentiation has previously been described in mouse chondrocytes with inactivated *Sox9* [31,32]. However, we did not detect significant difference in the expression of *Sox9* between the ATDC5-mutTUFT1, ATDC5-wtTUFT1 and ATDC5-ctrl cells suggesting that the described phenotype arises downstream from *Sox9* expression. We hypothesize that *TUFT1* overexpression inhibits differentiation signaling downstream from *Sox9* by altering cells’ calcium metabolism. This could further interfere the normal function of calcium-dependent adhesion molecule N-cadherin which is expressed in the early chondrogenesis following *Sox9* expression, but prior to expression of *Col2a1* and *Agc1* [33]. During chondrogenesis N-cadherin plays a role in cell aggregation [34,35] an event which has been proposed to be a crucial step in the early phase of the differentiation cascade, influencing also *COL2A1* expression *in vitro* in chick chondrocytes [36]. Assessing the effect of the identified mutation in relation to the function of N-cadherin could be an interesting topic for further studies.

This study has some limitations. Whole exome sequencing allows us to detect only the coding and surrounding region variants, while non-coding variations are missed. Regulatory regions locating in non-coding intronic and intergenic regions have been associated with OA (11) as well as with many other complex diseases [37–39]. However, it has been estimated that approximately 85% of mutations with large phenotypic effects are located on protein coding regions, and thus Mendelian traits result most often from exonic or splice-site mutations [40]. Another limitation is that only one family was available to the study. Unfortunately, population samples available from the same geographical area as the studied family, such as the Northern Finland Birth Cohorts (NFBC, http://www.oulu.fi/nfbc/), do not provide a suitable phenotype (radiographically determined arthritis of the first metatarsophalangeal joint) to perform a population level replication, nor does the chip-based genotype data from NFBC allow replication of rare variants, as they are excluded from the data for technical reasons. Disease symptoms limited to the MTP I joint may partially be explained by joint loading or site specific methylation profiles [41,42]. An animal model could inform more on tuftelin’s function during development of MTP I OA, but would be challenging to implement due the specific characteristics of human foot anatomy and impact of physical loading and environmental factors on the disease phenotype. A genome editing method, such as CRISPR-Cas9, would need to be applied to generate a mouse model with the specific *TUFT1* mutation, as *TUFT1* knockout mice do not display deformities in joints, bones or enamel [43]. A genome editing method could be also applied to generate an *in vitro* model that would help to better distinguish the effect of the identified mutation from *TUFT1* overexression.

Results of the present study implicate that *TUFT1* plays a role in chondrocyte differentiation and cartilage mineralization apparently on a calcium-related pathway, and thus can be considered as a novel susceptibility gene for MTP I OA. Our findings also advocate that investigation of rare variants and familial forms of complex phenotypes can provide valuable information about pathogenic mechanisms behind common diseases, such as OA. Further studies are needed to determine whether *TUFT1* variants associate to cartilage phenotypes on population level and to elucidate the precise biological function of tuftelin in chondrogenesis and mineralization of cartilage.

## Materials and methods

### Subjects

This study was approved by the Ethical Committee of the Northern Ostrobothnia Hospital District and a written consent was obtained from the study participants. Nissi *et al*. (2011) reported a Finnish family where early-onset MTP I OA is inherited in an autosomal dominant manner [15]. Thirteen out of 52 family members were affected (Fig 1). The mean age of onset was 26, ranging from 12 to 51. The radiological findings are described in detail by Nissi *et al*. [15]. Briefly, the X-ray images of the affected family members revealed typical OA related findings in the first MTP joint including joint space narrowing, subchondral sclerosis, osteophyte and cyst formation. Otherwise the other joints of the feet were healthy and there was no evidence of erosive lesions. No other deformities in the skeletal body were detected and large joints were not affected even in the older family members. Affected family members were otherwise healthy and had normal body height and weight. Also, individuals participating to the present study did not display any self-reported dental phenotype.

All the family members were contacted and interviewed. Four patients (II-4, III-1, III-3, III-12) having symptomatic and radiographic bilateral OA in addition to five asymptomatic family members (III-2, III-5, III-6, III-10, III-19) were willing to participate to the study.

### Whole exome sequencing

Three affected family members (II-4, III-1 and III-12) were selected for exome sequencing. In addition, two unrelated individuals were exome sequenced and used as control samples in the variant filtering steps. Blood samples were obtained from the participants and DNA was extracted using standard protocols. Exome sequencing and variant calling were done at the Institute for Molecular Medicine Finland (FIMM). Exonic sequences were enriched using the NimbleGen SeqCap EZ Human Exome Library v2.0 (Roche, NimbleGen, Inc., Madison, USA), and sequencing was done using a High Seq 2000 platform (Illumina, San Diego, USA). To ensure the quality of the variants, the exome data were taken through the FIMM bioinformatics pipeline [44].

### Variant annotation and filtering

Exome sequence data was filtered based on three assumptions. The causal variant was assumed to fit to the autosomal dominant model of inheritance as appeared in the pedigree. Furthermore, the causal variant(s) was/were presumed to be *de novo* or rare, since to our knowledge no similar families were previously described in the literature. Penetrance was assumed to be 100%, and therefore all the individuals having the disease genotype were presumed to be symptomatic. Variant filtering was done in R by selecting the variants shared by the affected family members and then excluding the variants found in the two unrelated control samples. Next, the variants previously not annotated into the Single Nucleotide Polymorphism Database 135 (dbSNP135), rare variants according to the 1000 Genomes Project (minor allele frequency ≤ 0.01) and variants with a record in dbSNP but with unknown frequency were annotated using ANNOVAR [45]. Exonic variants with harmful prediction by SIFT [46], PolyPhen 2 [47] or MutationTaster [48] algorithms and variants located at splice sites were considered harmful and chosen for further validation.

### Variant validation using Sanger sequencing

The selected variants were genotyped in all family members with DNA available using Sanger sequencing. Primers used in Sanger sequencing were designed using Primer3 v0.4.0 [49]. Primer sequences and PCR conditions are available on request. Purified PCR products were sequenced using ABI3500xL Genetic Analyzer and BigDye Terminator vs.1.1 reagents (Life Technologies, Carlsbad, USA). The results were analyzed using Variant Reporter version 2.0 (Life Technologies, Carlsbad, USA).

### Detection of CNVs

Detection of CNVs from whole exome data was done using a read count based CNV caller cn.MOPS [50]. CNVs regions shared by the samples were annotated using Database of Genomic Variants [51] to identify common CNVs.

### *TUFT1* constructs

A *TUFT1* cDNA clone was obtained from GenScript and cloned into pcDNA3.1 (-) expression vector (Invitrogen, Carlsbad, USA) using BamHI and XbaI restriction enzymes. The *TUFT1* variant c.524C>T was generated using QuikChange Site-Directed Mutagenesis Kit (Stratagene, San Diego, USA) according to the manufacturer’s instructions. All sequences were confirmed by capillary sequencing.

### Cell culture and transfections

HEK293 cells were cultured and maintained in DMEM with 10% FBS and plated in 10cm-plates at a density of 2 x 10^6^ cells/plate for protein analyses and in 6-well plates at a density of 3.5 x 10^5^ cells/well for real-time qPCR analyses 24h prior to transient transfections. Transfections for protein and qPCR analysis were performed using 5.6 μg DNA (wt or mutant *TUFT1* construct) and 40 μl FuGENE HD transfection reagent (Promega, Madison, USA) or 2 μg DNA and 14 μl FuGENE HD transfection reagent, respectively.

Mouse ATDC5 cells are widely used to model of chondrogenic differentiation and subsequent mineralization [52,53]. We applied a model for ATDC5 cells developed by Newton *et al*. allowing us to study chondrogenesis and ECM mineralization in a 15-day period [54]. ATDC5 cells (Sigma, St Louis, USA) were cultured in maintenance medium DMEM/F-12 (1:1) with GlutaMAX I (Gibco, Paisley, Scotland) containing 5% FBS, 1% sodium pyruvate, 0,1% penicillin (Sigma, St Louis, USA) and 0,01% Fungizone (Cambrex Bio Science, Walkersville, MD). For stable transfections 3.5×10^5^ cells/well were plated on six-well plates and transfected at a ratio of 7:2 FuGENE HD transfection reagent (Promega, Madison, USA) to 2 μl of DNA according to the manufacturer’s instructions. Empty pcDNA3.1 (-) was used as a control. After 24h the medium was replaced with fresh maintenance medium supplemented with 500 μg/ml Geneticin, G418 (Sigma, St Louis, USA). The selection medium was changed every second or third day until all of the cells without the neomycin resistance gene on the on a separate control plate were killed. Cell death was evaluated using light microscopy (Leica Microsystems, Wetzlar, Germany). A mixed population of stable G418 transfectants was expanded and cultured in differentiation medium (maintenance medium supplemented with 1x insulin-transferrin-selenium, 500 μg/ml Geneticin). The cells were plated in 24-well plates at a density of 5.0×10^4^ cells/ml. After the cells had reached confluency, 50 μg/ml ascorbic acid and 10 mM βGP were added and the cells were incubated in a humidified atmosphere (37°C, 5% CO2) for 15 days.

During differentiation chondrocytes are expected to form mineralizing cartilage nodules that can be detected as opaque deposits in microscopy images [54,55]. Nodule formation of ATDC5 was determined, and pictures were taken after 15 days of differentiation using EVOS fluorescence microscope.

### Real-time qPCR

Preceding to qPCR, total RNA was extracted from cells using an E.Z.N.A Total RNA Kit (Omega Bio-Tek, Norcross, USA) with RNase-free DNase (Omega Bio-Tek, Norcross, USA) treatment and cDNA was synthesized using 1 μg RNA per sample using an iScript cDNA Synthesis Kit (Bio-Rad, Hercules, USA). The effect of the c.524C>T on *TUFT1* expression was studied in HEK293 cells at one time point from three duplicate samples using an iTaq Universal SYBR Green Supermix kit (Bio-Rad, Hercules, USA) according to the manufacturer’s instructions in a CFX96 Real-Time System (Bio-Rad, Hercules, USA). ΔCt was determined by using *ACTB* (beta actin) and *B2M* (beta-2-microglobulin) as reference genes.

In chondrogenic differentiation mesenchymal progenitor cells proliferate and differentiate into chondrocytes. During this step, chondrocytes express *Sox9* transcription factor and number of cartilage matrix genes, including *Col2a1* and *Agc1*. This is followed by endochondral ossification when chondrocytes lose their differentiated phenotype, and became hypertrophic and mature chondrocytes. In this phase cells start to express matrix metallopeptidase 13, a cartilage degrading enzyme encoded by *Mmp13*, alkaline phosphatase (*Alpl*) which is an important enzyme for mineralization, runt related transcription factor 2 (*Runx2)* involved in regulation of skeletal gene expression and type X collagen (*Col10a1*), a specific marker for hypertrophic chondrocytes (reviewed in [56] and [57]). To analyze the expression of these chondrogenic and hypertrophic markers in chondrocytes, a real-time qPCR was carried out on four samples in duplicate on days 8, 12 and 15 and three technical repeats were performed. The gene expression levels were determined by the comparative C_T_ (2^-ΔΔCT^) method [58] using *Hprt* (hypoxanthine guanine phosphoribosyl transferase) and *Ppia* (peptidylprolyl isomerase A) as reference genes.

Oligonucleotide primers used in real-time qPCR amplification are listed in S4 Table. For each primer no template controls were included in duplicate.

### Preparation of cell lysates and Western blotting

After 48h of culturing, the growth media of HEK293 cells transiently transfected with wt or mutant *TUFT1* were collected (cell medium fraction, CM) and the cells were washed with ice cold phosphate buffered saline (PBS). Buffer 1 (10 mM Hepes (pH 7.9), 10 mM KCl, 1.5 mM MgCl2, 0.1 mM EDTA, 0.1 mM EGTA, 0.1% TritonX-100, 1 mM DTT, 1 × protease inhibitor (cOmplete ULTRA Tablets, mini, Roche, Mannheim, Germany)) was added to the plates and the cells were scraped off. Next, the cells were incubated 30 min with constant agitation at 4°C. The samples were centrifuged and the supernatants were collected (cytosolic fraction, CF). The pellet was washed with PBS and the cells were resuspended in buffer 2 (20 mM Hepes (pH 7.9), 400 mM NaCl, 0.25 mM EGTA, 1.5 mM MgCl2, 10% glycerol, 0.5 mM DTT, 1 × protease inhibitor (cOmplete ULTRA Tablets, mini, Roche, Mannheim, Germany)) and incubated a further 20 min with constant agitation at 4°C. The samples were centrifuged and supernatants collected (nuclear fraction, NF).

To analyze the effect of the c.524C>T on tuftelin expression and localization, 12.5% sodium dodecyl sulfate polyacrylamide gel electrophoresis (SDS-PAGE) gels were prepared. For electrophoresis 1 μg of total protein was analyzed under reducing conditions. Proteins separated on the gels were transferred onto polyvinylidene difluoride membranes (Millipore, Bedford, USA), which were blocked with 1×TBS (50 mM Tris-Cl, 150 mM NaCl, pH 7.5) with 0.05% Tween 20 (Merck, Darmstadt, Germany) containing 5% non-fat dry milk and probed with the primary antibody (1:2000 Anti-TUFT1 (86–100) antibody produced in rabbit (Sigma, St Louis, USA) in 1xTBS). Anti-beta Tubulin produced in rabbit (Abcam, Cambridge, UK) was used as a loading control. To visualize the immune complex 1:10 000 Anti-Rabbit IgG—Peroxidase antibody produced in goat in 1xTBS (Sigma, St Louis, USA) was used. The imaging of Western blot membranes was performed using Luminescent Image Analyzer LAS-3000 (FUJIFILM Medical Systems, USA) and the signal densities were quantified using ImageJ 1.47v (National Institutes of Health, USA).

### Histochemical staining of ATDC5 cells

Calcium and proteoglycan content of mixed populations of ATDC5-ctrl, ATDC5-wtTUFT1, and ATDC5-mutTUFT1 cells were determined by staining the cell layers with Alizarin red stain (Sigma, St Louis, USA) or Alcian blue stain (Sigma, St Louis, USA), respectively. Cells were grown 15 days after their mineralization state was analyzed. To evaluate calcium concentration, ATDC5 cells were fixed with 4% paraformaldehyde (PFA) for 5 min at 4°C, washed with PBS, stained with 2% Alizarin red (pH 4.2) for 5 min in room temperature, washed with distilled water and bound dye was extracted with 10% cetylpyridium chloride for 10 min. Optical density (OD) of the samples was determined at 570 nm by spectrophotometry. Proteoglycan content was analyzed by washing ATDC5 cells with PBS, fixing with 95% methanol for 20 min, staining with 1% Alcian blue 8GX (Sigma, St Louis, USA) in 0.1M HCl overnight and rinsing with distilled water. Cell cultures were extracted with 6M guanidine-HCl for 6h at room temperature and the released dye was evaluated by measuring the OD at 630 nm by spectrophotometer.

### Statistical analysis

Differences in *TUFT1* expression in HEK293 cells as well as in ECM mineralization characterized by Alizarin red Alcian blue stainings were analyzed using Student’s t-test. Differences in marker gene expressions between ATDC5-ctrl, ATDC5-wtTUFT1, and ATDC5-mutTUFT1 cells were determined using repeated measurements analysis of variance (ANOVA) followed by Tukey’s Honest Significant Difference (HSD) *post hoc* test. Prior to ANOVA and Tukey HSD, fold changes were log(x+1) transformed. P-values smaller than 0.05 were considered statistically significant in all the analyses. Statistical analyses were performed using Microsoft Excel 2013 (Student’s t-test for *TUFT1* expression in HEK293 cells) and R version 3.2.2. (repeated measurement ANOVA, Tukey HSD and Student’s t-test for ECM mineralization).

## Supporting information

S1 Fig The original uncropped Western blot image. 1 μg of total protein was used in the preparation of Western blot. Anti-TUFT1 and Anti-beta Tubulin primary antibodies and Anti-Rabbit IgG—Peroxidase secondary antibody were used in the experiment.
(TIF)

S1 Table Variants selected for validation by Sanger sequencing. (DOCX)

S2 Table Expression of marker genes for chondrocyte differentiation and hypertrophy (real-time qPCR): Repeated measurements ANOVA. Measurements were done separately for ATDC5-ctrl, ATDC5-wtTUFT1 and ATDC5-mutTUFT1 cell populations (CELL) at three time points (DAY). CELL:DAY interaction indicates whether the relative gene expression level changes over time in differing manner in the three cell populations.*P ≤ 0.05; ** P ≤ 0.01; *** P ≤ 0.001.
(DOCX)

S3 Table Expression of marker genes for chondrocyte differentiation and hypertrophy (real-time qPCR): Tukey HSD. Pairwise comparisons of the marker gene expression levels in the three cell populations at the three time points.
(DOCX)

S4 Table Primers used in qPCR analyses. (DOCX)

## References

[1] Johnson VL, Hunter DJ. The epidemiology of osteoarthritis. Best Pract Res Clin Rheumatol 2014 2;28(1):5–15. 10.1016/j.berh.2014.01.004 24792942

[2] Loeser RF, Goldring SR, Scanzello CR, Goldring MB. Osteoarthritis: a disease of the joint as an organ. Arthritis Rheum 2012 6;64(6):1697–1707. 10.1002/art.34453 22392533 PMC3366018

[3] Arden N, Nevitt MC. Osteoarthritis: epidemiology. Best Pract Res Clin Rheumatol 2006 2;20(1):3–25. 10.1016/j.berh.2005.09.007 16483904

[4] Kean WF, Kean R, Buchanan WW. Osteoarthritis: symptoms, signs and source of pain. Inflammopharmacology 2004;12(1):3–31. 10.1163/156856004773121347 15035776

[5] Goldring MB, Marcu KB. Cartilage homeostasis in health and rheumatic diseases. Arthritis Res Ther 2009;11(3):224 10.1186/ar2592 19519926 PMC2714092

[6] Karlson EW, Mandl LA, Aweh GN, Sangha O, Liang MH, Grodstein F. Total hip replacement due to osteoarthritis: the importance of age, obesity, and other modifiable risk factors. Am J Med 2003 2 1;114(2):93–98. 12586227 10.1016/s0002-9343(02)01447-x

[7] Spector TD, Cicuttini F, Baker J, Loughlin J, Hart D. Genetic influences on osteoarthritis in women: a twin study. BMJ 1996 4 13;312(7036):940–943. 8616305 10.1136/bmj.312.7036.940 PMC2350783

[8] Meulenbelt I, Bijkerk C, Breedveld FC, Slagboom PE. Genetic linkage analysis of 14 candidate gene loci in a family with autosomal dominant osteoarthritis without dysplasia. J Med Genet 1997 12;34(12):1024–1027. 9429149 10.1136/jmg.34.12.1024 PMC1051158

[9] Ingvarsson T, Stefansson SE, Gulcher JR, Jonsson HH, Jonsson H, Frigge ML, et al A large Icelandic family with early osteoarthritis of the hip associated with a susceptibility locus on chromosome 16p. Arthritis Rheum 2001 11;44(11):2548–2555. 11710711 10.1002/1529-0131(200111)44:11<2548::aid-art435>3.0.co;2-s

[10] Jakkula E, Melkoniemi M, Kiviranta I, Lohiniva J, Raina SS, Perala M, et al The role of sequence variations within the genes encoding collagen II, IX and XI in non-syndromic, early-onset osteoarthritis. Osteoarthritis Cartilage 2005 6;13(6):497–507. 10.1016/j.joca.2005.02.005 15922184

[11] Taipale M, Jakkula E, Kamarainen OP, Gao P, Skarp S, Barral S, et al Targeted re-sequencing of linkage region on 2q21 identifies a novel functional variant for hip and knee osteoarthritis. Osteoarthritis Cartilage 2016 4;24(4):655–663. 10.1016/j.joca.2015.10.019 26603474

[12] Iagnocco A, Rizzo C, Gattamelata A, Vavala C, Ceccarelli F, Cravotto E, et al Osteoarthritis of the foot: a review of the current state of knowledge. Med Ultrason 2013 3;15(1):35–40. 23486622 10.11152/mu.2013.2066.151.ai1ofr2

[13] Trivedi B, Marshall M, Belcher J, Roddy E. A systematic review of radiographic definitions of foot osteoarthritis in population-based studies. Osteoarthritis Cartilage 2010 8;18(8):1027–1035. 10.1016/j.joca.2010.05.005 20472083

[14] Bergin SM, Munteanu SE, Zammit GV, Nikolopoulos N, Menz HB. Impact of first metatarsophalangeal joint osteoarthritis on health-related quality of life. Arthritis Care Res (Hoboken) 2012 11;64(11):1691–1698. 22623156 10.1002/acr.21729

[15] Nissi R, Perhomaa M, Silvennoinen L, Halonen V, Rasila R, Winblad I, et al Hereditary isolated metatarsophalangeal arthritis. Scand J Rheumatol 2011 1;40(1):22–25. 10.3109/03009742.2010.495081 20858143

[16] Deutsch D, Palmon A, Fisher LW, Kolodny N, Termine JD, Young MF. Sequencing of bovine enamelin ("tuftelin") a novel acidic enamel protein. J Biol Chem 1991 8 25;266(24):16021–16028. 1874744

[17] Shaffer JR, Carlson JC, Stanley BO, Feingold E, Cooper M, Vanyukov MM, et al Effects of enamel matrix genes on dental caries are moderated by fluoride exposures. Hum Genet 2015 2;134(2):159–167. 10.1007/s00439-014-1504-7 25373699 PMC4293346

[18] MacDougall M, Simmons D, Dodds A, Knight C, Luan X, Zeichner-David M, et al Cloning, characterization, and tissue expression pattern of mouse tuftelin cDNA. J Dent Res 1998 12;77(12):1970–1978. 10.1177/00220345980770120401 9839784

[19] Mao Z, Shay B, Hekmati M, Fermon E, Taylor A, Dafni L, et al The human tuftelin gene: cloning and characterization. Gene 2001 11 28;279(2):181–196. 11733143 10.1016/s0378-1119(01)00749-1

[20] Saarikoski ST, Rivera SP, Hankinson O. Mitogen-inducible gene 6 (MIG-6), adipophilin and tuftelin are inducible by hypoxia. FEBS Lett 2002 10 23;530(1–3):186–190. 12387890 10.1016/s0014-5793(02)03475-0

[21] Mori Y, Chung UI, Tanaka S, Saito T. Determination of differential gene expression profiles in superficial and deeper zones of mature rat articular cartilage using RNA sequencing of laser microdissected tissue specimens. Biomed Res 2014;35(4):263–270. 25152035 10.2220/biomedres.35.263

[22] Leiser Y, Silverstein N, Blumenfeld A, Shilo D, Haze A, Rosenfeld E, et al The induction of tuftelin expression in PC12 cell line during hypoxia and NGF-induced differentiation. J Cell Physiol 2011 1;226(1):165–172. 10.1002/jcp.22318 20658530

[23] Oliveira FS, Bellesini LS, Defino HL, da Silva Herrero CF, Beloti MM, Rosa AL. Hedgehog signaling and osteoblast gene expression are regulated by purmorphamine in human mesenchymal stem cells. J Cell Biochem 2012 1;113(1):204–208. 10.1002/jcb.23345 21898541

[24] Park H, Kim JI, Ju YS, Gokcumen O, Mills RE, Kim S, et al Discovery of common Asian copy number variants using integrated high-resolution array CGH and massively parallel DNA sequencing. Nat Genet 2010 5;42(5):400–405. 10.1038/ng.555 20364138 PMC3329635

[25] Venselaar H, Te Beek TA, Kuipers RK, Hekkelman ML, Vriend G. Protein structure analysis of mutations causing inheritable diseases. An e-Science approach with life scientist friendly interfaces. BMC Bioinformatics 2010 11 8;11:548-2105-11-548. 10.1186/1471-2105-11-548 PMC2992548 21059217

[26] Burkhard P, Stetefeld J, Strelkov SV. Coiled coils: a highly versatile protein folding motif. Trends Cell Biol 2001 2;11(2):82–88. 11166216 10.1016/s0962-8924(00)01898-5

[27] Deutsch D, Leiser Y, Shay B, Fermon E, Taylor A, Rosenfeld E, et al The human tuftelin gene and the expression of tuftelin in mineralizing and nonmineralizing tissues. Connect Tissue Res 2002;43(2–3):425–434. 12489194 10.1080/03008200290001186

[28] Ea HK, Nguyen C, Bazin D, Bianchi A, Guicheux J, Reboul P, et al Articular cartilage calcification in osteoarthritis: insights into crystal-induced stress. Arthritis Rheum 2011 1;63(1):10–18. 10.1002/art.27761 20862682

[29] DeLise AM, Fischer L, Tuan RS. Cellular interactions and signaling in cartilage development. Osteoarthritis Cartilage 2000 9;8(5):309–334. 10.1053/joca.1999.0306 10966838

[30] Luo W, Wen X, Wang HJ, MacDougall M, Snead ML, Paine ML. In vivo overexpression of tuftelin in the enamel organic matrix. Cells Tissues Organs 2004;177(4):212–220. 10.1159/000080134 15459477

[31] Bi W, Deng JM, Zhang Z, Behringer RR, de Crombrugghe B. Sox9 is required for cartilage formation. Nat Genet 1999 5;22(1):85–89. 10.1038/8792 10319868

[32] de Crombrugghe B, Lefebvre V, Behringer RR, Bi W, Murakami S, Huang W. Transcriptional mechanisms of chondrocyte differentiation. Matrix Biol 2000 9;19(5):389–394. 10980415 10.1016/s0945-053x(00)00094-9

[33] Stanton LA, Underhill TM, Beier F. MAP kinases in chondrocyte differentiation. Dev Biol 2003 11 15;263(2):165–175. 14597193 10.1016/s0012-1606(03)00321-x

[34] Oberlender SA, Tuan RS. Expression and functional involvement of N-cadherin in embryonic limb chondrogenesis. Development 1994 1;120(1):177–187. 8119125 10.1242/dev.120.1.177

[35] Delise AM, Tuan RS. Analysis of N-cadherin function in limb mesenchymal chondrogenesis in vitro. Dev Dyn 2002 10;225(2):195–204. 10.1002/dvdy.10151 12242719

[36] Tacchetti C, Tavella S, Dozin B, Quarto R, Robino G, Cancedda R. Cell condensation in chondrogenic differentiation. Exp Cell Res 1992 5;200(1):26–33. 1563490 10.1016/s0014-4827(05)80067-9

[37] Gaulton KJ, Nammo T, Pasquali L, Simon JM, Giresi PG, Fogarty MP, et al A map of open chromatin in human pancreatic islets. Nat Genet 2010 3;42(3):255–259. 10.1038/ng.530 20118932 PMC2828505

[38] Musunuru K, Strong A, Frank-Kamenetsky M, Lee NE, Ahfeldt T, Sachs KV, et al From noncoding variant to phenotype via SORT1 at the 1p13 cholesterol locus. Nature 2010 8 5;466(7307):714–719. 10.1038/nature09266 20686566 PMC3062476

[39] Westra HJ, Peters MJ, Esko T, Yaghootkar H, Schurmann C, Kettunen J, et al Systematic identification of trans eQTLs as putative drivers of known disease associations. Nat Genet 2013 10;45(10):1238–1243. 10.1038/ng.2756 24013639 PMC3991562

[40] Majewski J, Schwartzentruber J, Lalonde E, Montpetit A, Jabado N. What can exome sequencing do for you? J Med Genet 2011 9;48(9):580–589. 10.1136/jmedgenet-2011-100223 21730106

[41] Schibler L, Gibbs L, Benoist-Lasselin C, Decraene C, Martinovic J, Loget P, et al New insight on FGFR3-related chondrodysplasias molecular physiopathology revealed by human chondrocyte gene expression profiling. PLoS One 2009 10 29;4(10):e7633 10.1371/journal.pone.0007633 19898608 PMC2764091

[42] Smith R. Mechanical loading effects on articular cartilage matrix metabolism and osteoarthritis In: Buckwalter JA, Lotz MK & Stolz JF (eds) Osteoarthritis, Inflammation and Degradation: A Continuum. 2007;Amsterdam, IOS Press: 14–23.

[43] Eppig JT, Blake JA, Bult CJ, Kadin JA, Richardson JE, Mouse Genome Database Group. The Mouse Genome Database (MGD): facilitating mouse as a model for human biology and disease. Nucleic Acids Res 2015 1;43(Database issue):D726–36. 10.1093/nar/gku967 25348401 PMC4384027

[44] Sulonen AM, Ellonen P, Almusa H, Lepisto M, Eldfors S, Hannula S, et al Comparison of solution-based exome capture methods for next generation sequencing. Genome Biol 2011 9 28;12(9):R94-2011-12-9-r94. 10.1186/gb-2011-12-9-r94 PMC3308057 21955854

[45] Wang K, Li M, Hakonarson H. ANNOVAR: functional annotation of genetic variants from high-throughput sequencing data. Nucleic Acids Res 2010 9;38(16):e164 10.1093/nar/gkq603 20601685 PMC2938201

[46] Ng PC, Henikoff S. Predicting deleterious amino acid substitutions. Genome Res 2001 5;11(5):863–874. 10.1101/gr.176601 11337480 PMC311071

[47] Adzhubei IA, Schmidt S, Peshkin L, Ramensky VE, Gerasimova A, Bork P, et al A method and server for predicting damaging missense mutations. Nat Methods 2010 4;7(4):248–249. 10.1038/nmeth0410-248 20354512 PMC2855889

[48] Schwarz JM, Rodelsperger C, Schuelke M, Seelow D. MutationTaster evaluates disease-causing potential of sequence alterations. Nat Methods 2010 8;7(8):575–576. 10.1038/nmeth0810-575 20676075

[49] Koressaar T, Remm M. Enhancements and modifications of primer design program Primer3. Bioinformatics 2007 5 15;23(10):1289–1291. 10.1093/bioinformatics/btm091 17379693

[50] Klambauer G, Schwarzbauer K, Mayr A, Clevert DA, Mitterecker A, Bodenhofer U, et al cn.MOPS: mixture of Poissons for discovering copy number variations in next-generation sequencing data with a low false discovery rate. Nucleic Acids Res 2012 5;40(9):e69 10.1093/nar/gks003 22302147 PMC3351174

[51] MacDonald JR, Ziman R, Yuen RK, Feuk L, Scherer SW. The Database of Genomic Variants: a curated collection of structural variation in the human genome. Nucleic Acids Res 2014 1;42(Database issue):D986–92. 10.1093/nar/gkt958 24174537 PMC3965079

[52] Shukunami C, Ishizeki K, Atsumi T, Ohta Y, Suzuki F, Hiraki Y. Cellular hypertrophy and calcification of embryonal carcinoma-derived chondrogenic cell line ATDC5 in vitro. J Bone Miner Res 1997 8;12(8):1174–1188. 10.1359/jbmr.1997.12.8.1174 9258747

[53] Fujita T, Meguro T, Izumo N, Yasutomi C, Fukuyama R, Nakamuta H, et al Phosphate stimulates differentiation and mineralization of the chondroprogenitor clone ATDC5. Jpn J Pharmacol 2001 3;85(3):278–281. 11325020 10.1254/jjp.85.278

[54] Newton PT, Staines KA, Spevak L, Boskey AL, Teixeira CC, Macrae VE, et al Chondrogenic ATDC5 cells: an optimised model for rapid and physiological matrix mineralisation. Int J Mol Med 2012 11;30(5):1187–1193. 10.3892/ijmm.2012.1114 22941229 PMC3573767

[55] Shukunami C, Ishizeki K, Atsumi T, Ohta Y, Suzuki F, Hiraki Y. Cellular hypertrophy and calcification of embryonal carcinoma-derived chondrogenic cell line ATDC5 in vitro. J Bone Miner Res 1997 8;12(8):1174–1188. 10.1359/jbmr.1997.12.8.1174 9258747

[56] Kronenberg HM. Developmental regulation of the growth plate. Nature 2003 5 15;423(6937):332–336. 10.1038/nature01657 12748651

[57] Mackie EJ, Ahmed YA, Tatarczuch L, Chen KS, Mirams M. Endochondral ossification: how cartilage is converted into bone in the developing skeleton. Int J Biochem Cell Biol 2008;40(1):46–62. 10.1016/j.biocel.2007.06.009 17659995

[58] Schmittgen TD, Livak KJ. Analyzing real-time PCR data by the comparative C(T) method. Nat Protoc 2008;3(6):1101–1108. 18546601 10.1038/nprot.2008.73

