# Supplementary figures and images for: TUFT1, a novel candidate gene for metatarsophalangeal osteoarthritis, plays a role in chondrogenesis on a calcium-related pathway
Source: PLoS One. 2017 Apr 14;12(4):e0175474. doi: 10.1371/journal.pone.0175474 (PMC5391938; doi:10.1371/journal.pone.0175474)

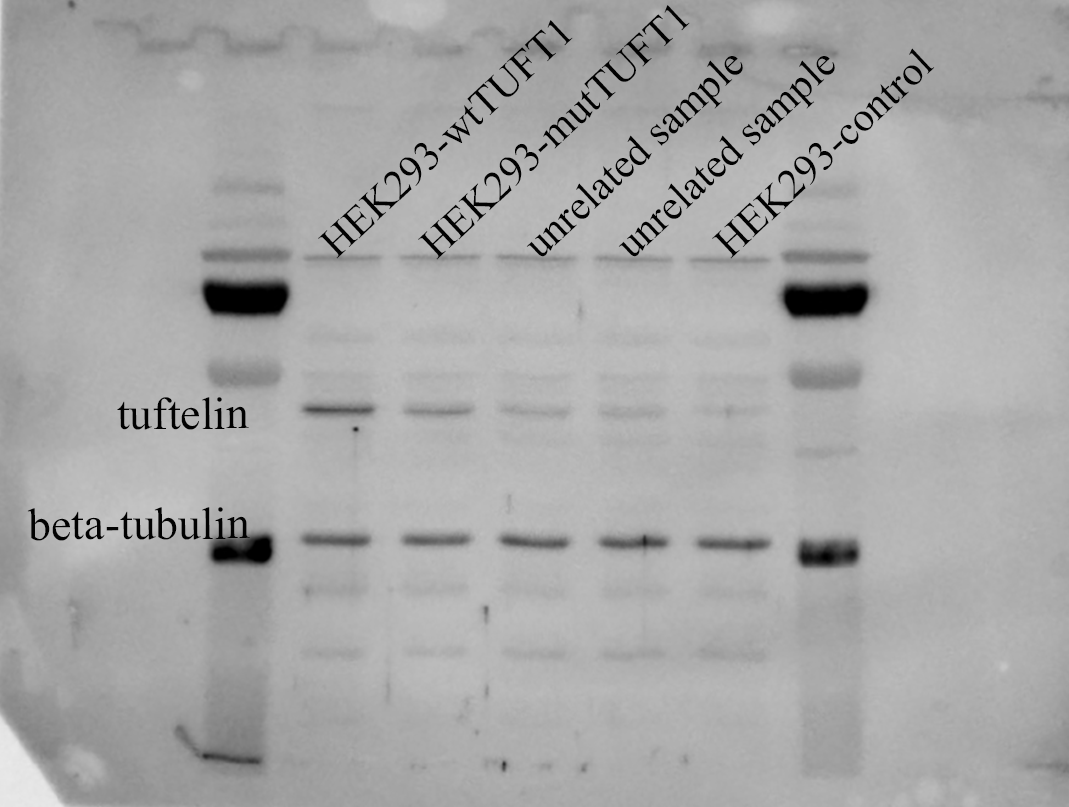

Supplement: S1 Fig — 1 μg of total protein was used in the preparation of Western blot. Anti-TUFT1 and Anti-beta Tubulin primary antibodies and Anti-Rabbit IgG—Peroxidase secondary antibody were used in the experiment. (TIF) [file pone.0175474.s001.tif]
