# Supplementary material for: TUFT1, a novel candidate gene for metatarsophalangeal osteoarthritis, plays a role in chondrogenesis on a calcium-related pathway
Source: PLoS One. 2017 Apr 14;12(4):e0175474. doi: 10.1371/journal.pone.0175474 (PMC5391938; doi:10.1371/journal.pone.0175474)
Supplement: S1 Table — (DOCX) [file pone.0175474.s002.docx]

**S1 Fig. The original uncropped Western blot image.** 1 µg of total protein was used in preparation of Western blot. Anti-TUFT1 and Anti-beta Tubulin primary antibodies and Anti-Rabbit IgG –Peroxidase secondary antibody were used in the experiment.

**S1 Table. Variants selected for validation by Sanger sequencing**

| **Genes** | **Chr** | **Locus** | **dsSNP135** | **MAF**  **(1000G)** | **Function** | ***In silico* analysis** | | |
| --- | --- | --- | --- | --- | --- | --- | --- | --- |
|  |  |  |  |  |  | **SIFT** | **PP2** | **MT** |
| *IFFO2* | 1 | 19236932 | rs186558947 | 0.0014 | ns SNV | 0.00 (d) | 0.998 (d) | 0.966 (d) |
| *IGSF3* | 1 | 117142700 | rs112943111 | NA | ns SNV | 0.09 | 1.000 (d) | 1.000 (d) |
| *NBPF8* | 1 | 148023040 | rs113502897 | NA | unknown | 0.05 (d) | 1.000 (d) | 1.000 (d) |
| *TUFT1* | 1 | 151542176 | rs41310883 | 0.01 | ns SNV | 0.17 | 1.000 (d) | 0.058 |
| *URB2* | 1 | 229787055 | rs41310553 | 0.01 | ns SNV | NA | 1.000 (d) | 0.983 (d) |
| *SP110* | 2 | 231042873 | rs149485401 | 0.01 | ns SNV | NA | 1.000 (d) | 0.868 (d) |
| *COL6A3* | 2 | 238280504 | rs146092501 | 0.0027 | ns SNV | 0.38 | 0.994 (d) | 0.964 (d) |
| *HHATL* | 3 | 42740576 | rs145469527 | 0.0023 | ns SNV | 0.52 | 0.017 | 0.990 (d) |
| *KIF9* | 3 | 47308746 | rs115200348 | 0.01 | ns SNV | 0.01 (d) | 0.969 (d) | 0.996 (d) |
| *SYNPR* | 3 | 63264392 | NA | NA | ns SNV | 0.00 (d) | 0.991 (d) | 0.289 |
| *ZNF717* | 3 | 75786919 | rs77101176 | NA | ns SNV | 0.00 (d) | 0.998 (d) | NA |
| *VEGFC* | 4 | 177650866 | rs41278571 | 0.01 | ns SNV | 0.00 (d) | 1.000 (d) | 0.993 (d) |
| *HLA-DRB1* | 6 | 32548026 | rs9269744 | NA | ns SNV | 0.07 | 1.000 (d) | 0.668 |
| *RSPH4A* | 6 | 116949359 | rs117169123 | 0.01 | ns SNV | 0.89 | 0.842 | 0.965 (d) |
| *FAM20C* | 7 | 286383 | NA | NA | ns SNV | 0.32 | 0.998 (d) | NA |
| *GBGT1* | 9 | 136031315 | rs117595304 | 0.0014 | ns SNV | 0.05 (d) | 0.999 (d) | 0.967 (d) |
| *OR13C2* | 9 | 107367393-107367396 | rs143198170 | NA | frameshift deletion | damaging | NA | disease causing |
| *LRIT2* | 10 | 85985194 | rs12773843 | 0.0023 | ns SNV | 0.00 (d) | 1.000 (d) | 0.994 (d) |
| *TACC2* | 10 | 123846421 | rs142508761 | 0.0018 | ns SNV | 0.02 (d) | 0.058 | NA |
| *OR5P2* | 11 | 7818384 | NA | NA | stopgain insertion | damaging | NA | disease causing |
| *PIBF1* | 13 | 73409509 | COSM253063 | NA | splicing insertion | NA | NA | NA |
| *OR11H12* | 14 | 19378312 | rs61969158 | NA | ns SNV | 0.00 (d) | 0.955 (d) | 0.009 |
| *C14orf159* | 14 | 91671115 | rs149609125 | 0.0005 | ns SNV | 0.03 (d) | 0.999 (d) | 0.046 |
| *PCSK6* | 15 | 101845482 | rs34631529 | NA | unknown | 0.00 (d) | 1.000 (d) | 0.948 (d) |
| *HYDIN* | 16 | 70896033 | rs1626593 | NA | ns SNV | 0.01 (d) | 0.999 (d) | 0.396 |
| *C16orf3* | 16 | 90095482 | rs76688449 | 0.01 | ns SNV | NA | 0.995 (d) | 0.008 |
| *RCVRN* | 17 | 9801447 | NA | NA | ns SNV | 0.05 (d) | 0.997 (d) | 0.975 (d) |
| *MYH2* | 17 | 10442777 | NA | 0.0005 | ns SNV | 0.00 (d) | 0.999 (d) | 0.332 |
| *KRTAP4-4* | 17 | 39316930 | rs111435962 | 0.0023 | ns SNV | 0.00 (d) | 1.000 (d) | 0.931 (d) |
| *KLHL11* | 17 | 40010572 | rs140333312 | 0.0037 | ns SNV | 0.00 (d) | 0.023 | 0.494 |
| *MBD3L3* | 19 | 7056571 | rs111605618 | NA | ns SNV | 0.14 | NA | 0.929 (d) |
| *ZNF571* | 19 | 38056087 | rs45542635 | 0.01 | ns SNV | 0.03 (d) | 0.999 (d) | 0.037 |
| *CYP2F1* | 19 | 41622108 | rs3833221 | NA | frameshift insertion | damaging | NA | disease causing |
| *ZNF415* | 19 | 53612490 | rs150851211 | 0.0014 | ns SNV | 0.00 (d) | 0.999 (d) | 0.002 |
| *LILRB5* | 19 | 54761023 | rs150778096 | NA | ns SNV | NA | 0.999 (d) | 0.051 |
| *KIR3DL1* | 19 | 55329934 | rs62124092 | NA | ns SNV | 0.01 (d) | 0.868 (d) | 0.006 |
| *KIR3DL1* | 19 | 55330036 | rs643861 | NA | ns SNV | 0.02 (d) | 0.952 (d) | 0.000 |
| *CPNE1* | 20 | 34215235 | NA | NA | frameshift insertion | damaging | NA | polymorphism |

Chr,chromosome; 1000G, The 1000 Genome Project; SIFT, Sorting Intolerant From Tolerant; PP2, Polymorphism Phenotyping v2 (PolyPhen2); MT, MutationTaster; ns SNV, nonsynonymoys single nucleotide variant; NA, not available; d, damaging prediction (cutoff values SIFT ≤ 0.05, PP2 and MT ≥ 0.85).
