# Supplementary material for: TUFT1, a novel candidate gene for metatarsophalangeal osteoarthritis, plays a role in chondrogenesis on a calcium-related pathway
Source: PLoS One. 2017 Apr 14;12(4):e0175474. doi: 10.1371/journal.pone.0175474 (PMC5391938; doi:10.1371/journal.pone.0175474)
Supplement: S2 Table — Measurements were done separately for ATDC5-ctrl, ATDC5-wtTUFT1 and ATDC5-mutTUFT1 cell populations (CELL) at three time points (DAY). CELL:DAY interaction indicates whether the relative gene expression level changes over time in differing manner in the three cell populations.*P ≤ 0.05; ** P ≤ 0.01; *** P ≤ 0.001. (DOCX) [file pone.0175474.s003.docx]

**S2 Table. Expression of marker genes for chondrocyte differentiation and hypertrophy (real-time qPCR): Repeated measurements ANOVA.** Measurements were done separately for ATDC5-ctrl, ATDC5-wtTUFT1 and ATDC5-mutTUFT1 cell populations (CELL) at three time points (DAY). CELL:DAY interaction indicates whether the relative gene expression level changes over time in differing manner in the three cell populations.

|  |  | **Degrees of freedom** | **Sum of squares** | **Mean square** | **F value** | **Pr(>F)** | **Significance** |
| --- | --- | --- | --- | --- | --- | --- | --- |
| *Sox9* | CELL | 2 | 0.191 | 0.095 | 1.053 | 0.354 |  |
|  | DAY | 2 | 1.992 | 0.996 | 11.004 | 6.55E-05 | *** |
|  | CELL:DAY | 4 | 0.330 | 0.083 | 0.913 | 0.461 |  |
|  | Residuals | 74 | 6.697 | 0.091 |  |  |  |
| *Col2a1* | CELL | 2 | 3.106 | 1.553 | 25.851 | 3.06E-09 | *** |
|  | DAY | 2 | 0.122 | 0.061 | 1.018 | 0.366 |  |
|  | CELL:DAY | 4 | 7.523 | 1.881 | 31.308 | 2.95E-15 | *** |
|  | Residuals | 74 | 4.446 | 0.060 |  |  |  |
| *Agc1* | CELL | 2 | 0.003 | 0.002 | 14.682 | 4.26E-06 | *** |
|  | DAY | 2 | 0.000 | 0.000 | 2.161 | 0.122 |  |
|  | CELL:DAY | 4 | 0.010 | 0.002 | 23.211 | 1.86E-12 | *** |
|  | Residuals | 74 | 0.008 | 0.000 |  |  |  |
| *Runx2* | CELL | 2 | 0.007 | 0.003 | 1.092 | 0.341 |  |
|  | DAY | 2 | 0.107 | 0.054 | 17.812 | 4.84E-07 | *** |
|  | CELL:DAY | 4 | 0.006 | 0.002 | 0.532 | 0.712 |  |
|  | Residuals | 74 | 0.223 | 0.003 |  |  |  |
| *Col10a1* | CELL | 2 | 0.000 | 0.000 | 3.840 | 0.0259 | * |
|  | DAY | 2 | 0.001 | 0.001 | 18.978 | 2.22E-07 | *** |
|  | CELL:DAY | 4 | 0.000 | 0.000 | 3.667 | 0.00884 | ** |
|  | Residuals | 74 | 0.002 | 0.000 |  |  |  |
| *Mmp13* | CELL | 2 | 1.09E-05 | 5.44E-06 | 12.786 | 1.70E-05 | *** |
|  | DAY | 2 | 1.21E-05 | 6.05E-06 | 14.225 | 5.92E-06 | *** |
|  | CELL:DAY | 4 | 4.37E-06 | 1.09E-06 | 2.568 | 0.045 | * |
|  | Residuals | 74 | 3.15E-05 | 4.25E-07 |  |  |  |
| *Alpl* | CELL | 2 | 0.027 | 0.013 | 1.366 | 0.26156 |  |
|  | DAY | 2 | 0.191 | 0.095 | 9.709 | 1.8E-04 | *** |
|  | CELL:DAY | 4 | 0.394 | 0.099 | 10.034 | 1.52E-06 | *** |
|  | Residuals | 74 | 0.727 | 0.010 |  |  |  |
| *TUFT1* | CELL | 2 | 8.40E-04 | 4.20E-04 | 219,415 | 2,00E-16 | *** |
|  | DAY | 2 | 4.70E-05 | 2.35E-05 | 12,275 | 2,49E-05 | *** |
|  | CELL:DAY | 4 | 1.93E-05 | 4.80E-06 | 2,519 | 0,0483 | * |
|  | Residuals | 74 | 1.42E-04 | 1.90E-06 |  |  |  |

*P ≤ 0.05; ** P ≤ 0.01; *** P ≤ 0.001
