# Supplementary material for: TUFT1, a novel candidate gene for metatarsophalangeal osteoarthritis, plays a role in chondrogenesis on a calcium-related pathway
Source: PLoS One. 2017 Apr 14;12(4):e0175474. doi: 10.1371/journal.pone.0175474 (PMC5391938; doi:10.1371/journal.pone.0175474)
Supplement: S3 Table — Pairwise comparisons of the marker gene expression levels in the three cell populations at the three time points. (DOCX) [file pone.0175474.s004.docx]

**S3 Table. Expression of marker genes for chondrocyte differentiation and hypertrophy (real-time qPCR): Tukey HSD.** Pairwise comparisons of the marker gene expression levels in the three cell populations at the three time points.

| **Gene** | **Day** | **Comparison** | **Difference in log (fold change+1)** | **95% Confidence Level** | | **P-value** | **Significance** |
| --- | --- | --- | --- | --- | --- | --- | --- |
| ***Sox9*** | **D8** | **mut-control** | 0.15408 | -0.30188 | 0.61004 | 0.97541 |  |
|  |  | **wt-control** | 0.20919 | -0.24678 | 0.66515 | 0.86734 |  |
|  |  | **wt-mut** | 0.05511 | -0.37478 | 0.48499 | 0.99998 |  |
|  | **D12** | **mut-control** | -0.19950 | -0.66659 | 0.26759 | 0.90722 |  |
|  |  | **wt-control** | -0.08236 | -0.53833 | 0.37360 | 0.99967 |  |
|  |  | **wt-mut** | 0.11714 | -0.32453 | 0.55881 | 0.99488 |  |
|  | **D15** | **mut-control** | -0.04497 | -0.50093 | 0.41099 | 1.00000 |  |
|  |  | **wt-control** | 0.12692 | -0.32904 | 0.58288 | 0.99290 |  |
|  |  | **wt-mut** | 0.17189 | -0.25800 | 0.60178 | 0.93470 |  |
| ***Col2a1*** | **D8** | **mut-control** | 0.50981 | 0.13831 | 0.88132 | 0.00120 | ** |
|  |  | **wt-control** | 0.50553 | 0.13402 | 0.87703 | 0.00136 | ** |
|  |  | **wt-mut** | -0.00428 | -0.35454 | 0.34597 | 1.00000 |  |
|  | **D12** | **mut-control** | -0.78815 | -1.16872 | -0.40759 | 0.00000 | *** |
|  |  | **wt-control** | -0.35742 | -0.72892 | 0.01408 | 0.06861 |  |
|  |  | **wt-mut** | 0.43073 | 0.07088 | 0.79059 | 0.00787 | ** |
|  | **D15** | **mut-control** | -1.19143 | -1.56293 | -0.81992 | 0.00000 | *** |
|  |  | **wt-control** | -0.83695 | -1.20845 | -0.46544 | 0.00000 | *** |
|  |  | **wt-mut** | 0.35448 | 0.00422 | 0.70474 | 0.04508 | * |
| ***Agc1*** | **D8** | **mut-control** | 0.01950 | 0.00386 | 0.03515 | 0.00470 | ** |
|  |  | **wt-control** | 0.02040 | 0.00475 | 0.03604 | 0.00255 | ** |
|  |  | **wt-mut** | 0.00089 | -0.01385 | 0.01564 | 1.00000 |  |
|  | **D12** | **mut-control** | -0.02610 | -0.04213 | -0.01008 | 0.00006 | *** |
|  |  | **wt-control** | -0.01860 | -0.03424 | -0.00295 | 0.00855 | ** |
|  |  | **wt-mut** | 0.00751 | -0.00764 | 0.02266 | 0.81039 |  |
|  | **D15** | **mut-control** | -0.04000 | -0.05564 | -0.02435 | 0.00000 | *** |
|  |  | **wt-control** | -0.03219 | -0.04783 | -0.01654 | 0.00000 | *** |
|  |  | **wt-mut** | 0.00781 | -0.00694 | 0.02256 | 0.74937 |  |
| ***Runx2*** | **D8** | **mut-control** | 0.01371 | -0.06941 | 0.09682 | 0.99983 |  |
|  |  | **wt-control** | 0.02172 | -0.06139 | 0.10483 | 0.99537 |  |
|  |  | **wt-mut** | 0.00801 | -0.07035 | 0.08637 | 1.00000 |  |
|  | **D12** | **mut-control** | -0.02980 | -0.11494 | 0.05534 | 0.96957 |  |
|  |  | **wt-control** | 0.00051 | -0.08260 | 0.08363 | 1.00000 |  |
|  |  | **wt-mut** | 0.03032 | -0.05019 | 0.11082 | 0.95332 |  |
|  | **D15** | **mut-control** | -0.03455 | -0.11766 | 0.04856 | 0.91950 |  |
|  |  | **wt-control** | -0.00926 | -0.09237 | 0.07385 | 0.99999 |  |
|  |  | **wt-mut** | 0.02529 | -0.05307 | 0.10365 | 0.98150 |  |
| ***Col10a1*** | **D8** | **mut-control** | 0.00553 | -0.00291 | 0.01397 | 0.48585 |  |
|  |  | **wt-control** | 0.00396 | -0.00448 | 0.01240 | 0.85239 |  |
|  |  | **wt-mut** | -0.00157 | -0.00952 | 0.00639 | 0.99937 |  |
|  | **D12** | **mut-control** | 0.00094 | -0.00770 | 0.00959 | 0.99999 |  |
|  |  | **wt-control** | 0.00375 | -0.00469 | 0.01219 | 0.88684 |  |
|  |  | **wt-mut** | 0.00281 | -0.00537 | 0.01098 | 0.97294 |  |
|  | **D15** | **mut-control** | -0.00690 | -0.01534 | 0.00154 | 0.19888 |  |
|  |  | **wt-control** | 0.00271 | -0.00573 | 0.01115 | 0.98203 |  |
|  |  | **wt-mut** | 0.00961 | 0.00166 | 0.01757 | 0.00702 | ** |
| ***Mmp13*** | **D8** | **mut-control** | -0.00024 | -0.00123 | 0.00075 | 0.99736 |  |
|  |  | **wt-control** | -0.00046 | -0.00145 | 0.00052 | 0.85145 |  |
|  |  | **wt-mut** | -0.00023 | -0.00116 | 0.00071 | 0.99721 |  |
|  | **D12** | **mut-control** | -0.00031 | -0.00132 | 0.00070 | 0.98652 |  |
|  |  | **wt-control** | -0.00055 | -0.00154 | 0.00044 | 0.70075 |  |
|  |  | **wt-mut** | -0.00024 | -0.00119 | 0.00072 | 0.99682 |  |
|  | **D15** | **mut-control** | -0.00115 | -0.00214 | -0.00016 | 0.01093 | * |
|  |  | **wt-control** | -0.00169 | -0.00268 | -0.00070 | 0.00002 | *** |
|  |  | **wt-mut** | -0.00054 | -0.00147 | 0.00039 | 0.65247 |  |
| ***Alpl*** | **D8** | **mut-control** | 0.18571 | 0.03551 | 0.33590 | 0.00524 | ** |
|  |  | **wt-control** | 0.13136 | -0.01884 | 0.28155 | 0.13459 |  |
|  |  | **wt-mut** | -0.05435 | -0.19595 | 0.08726 | 0.94799 |  |
|  | **D12** | **mut-control** | -0.08603 | -0.23989 | 0.06783 | 0.69052 |  |
|  |  | **wt-control** | -0.03024 | -0.18044 | 0.11995 | 0.99927 |  |
|  |  | **wt-mut** | 0.05579 | -0.08970 | 0.20127 | 0.94824 |  |
|  | **D15** | **mut-control** | -0.22844 | -0.37864 | -0.07825 | 0.00021 | *** |
|  |  | **wt-control** | -0.11564 | -0.26584 | 0.03455 | 0.26835 |  |
|  |  | **wt-mut** | 0.11280 | -0.02880 | 0.25441 | 0.22804 |  |
| ***TUFT1*** | **D8** | **mut-control** | 0.00170 | -0.00040 | 0.00380 | 0.20745 |  |
|  |  | **wt-control** | 0.00682 | 0.00472 | 0.00891 | 0.00000 | *** |
|  |  | **wt-mut** | 0.00512 | 0.00314 | 0.00709 | 0.00000 | *** |
|  | **D12** | **mut-control** | 0.00168 | -0.00046 | 0.00383 | 0.24676 |  |
|  |  | **wt-control** | 0.00736 | 0.00526 | 0.00946 | 0.00000 | *** |
|  |  | **wt-mut** | 0.00568 | 0.00365 | 0.00771 | 0.00000 | *** |
|  | **D15** | **mut-control** | 0.00415 | 0.00205 | 0.00625 | 0.00000 | *** |
|  |  | **wt-control** | 0.00882 | 0.00673 | 0.01092 | 0.00000 | *** |
|  |  | **wt-mut** | 0.00467 | 0.00270 | 0.00665 | 0.00000 | *** |

*P ≤ 0.05; ** P ≤ 0.01; *** P ≤ 0.001; mut, ATDC5-mutTUFT1; wt, ATDC5-mutTUFT1; control, ATDC5-ctrl
