# Supplementary material for: TUFT1, a novel candidate gene for metatarsophalangeal osteoarthritis, plays a role in chondrogenesis on a calcium-related pathway
Source: PLoS One. 2017 Apr 14;12(4):e0175474. doi: 10.1371/journal.pone.0175474 (PMC5391938; doi:10.1371/journal.pone.0175474)
Supplement: S4 Table — (DOCX) [file pone.0175474.s005.docx]

**S4 Table. Primers used in qPCR analyses.**

| Gene | Forward sequence | Reverse sequence | Amplicon [bp] | Access No.[RefSeq] | Reference |
| --- | --- | --- | --- | --- | --- |
| *TUFT1* | AAAGGACGCCACCATCCAG | GTGCTGAAGTTGCCATGACTG | 123 | NM_020127.2 | this study |
| *ACTB* | ACTCTTCCAGCCTTCCTTCC | CGTACAGGTCTTTGCGGATG | 104 | NM_001101.3 | this study |
| *B2M* | ACTCTCTCTTTCTGGCCTGG | ATGTCGGATGGATGAAACCC | 129 | NM_004048.2 | this study |
| *Col2a1* | AGGGCAACAGCAGGTTCACATAC | TGTCCACACCAAATTCCTGTTCA | 171 | NM_031163.3 | Aro *et al. 2012* |
| *Agc1* | CCAAACCAGCCTGACAACTT | TCTAGCATGCTCCACCACTG | 165 | NM_007424.2 | Aro *et al. 2012* |
| *Sox9* | ACGGCTCCAGCAAGAACAAG | TTGTGCAGATGCGGGTACTG | 109 | NM_011448.4 | Hattori *et al.* 2010 |
| *Mmp13* | CGATGAAGACCCCAACCCTAA | ACTGGTAATGGCATCAAGGGATA | 79 | NM_008607.2 | Caron *et al.* 2013 |
| *Alpl* | CCAACTCTTTTGTGCCAGAGA | GGCTACATTGGTGTTGAGCTTTT | 110 | NM_007431.3 | Zhang *et. al* 2011 |
| *Runx2* | ACCCAGCCACCTTTACCTAC | TATGGAGTGCTGCTGGTCTG | 151 | NM_001146038.2 | Gu *et al.* 2014 |
| *Col10a1* | TTCTGCTGCTAATGTTCTTGACC | GGGATGAAGTATTGTGTCTTGGG | 115 | NM_009925.4 | Seriwatanachai *et al.* 2012 |
| *Hprt* | CTGGTGAAAAGGACCTCTCGAA | CTGAAGTACTCATTATAGTCAAGGGCAT | 110 | NM_013556.2 | this study |
| *Ppia* | CGCGTCTCCTTCGAGCTGTTTG | TGTAAAGTCACCACCCTGGCACAT | 150 | NM_008907.1 | Mamo *et al.* 2007 |

Primer efficiency was determined from slope and is available by request**.**
